# The forehead is a better site than the sternum to check transcutaneous bilirubin during phototherapy in sick infants

**DOI:** 10.1186/s12887-020-02450-w

**Published:** 2020-12-05

**Authors:** Jaesung Jeon, Gina Lim, Ki Won Oh, Na Mi Lee, Hye Won Park, Mi Lim Chung

**Affiliations:** 1 Department of Pediatrics, Ulsan University Hospital, University of Ulsan College of Medicine, 877 Bangeojinsunhwan-doro, Dong-gu, 44033 Ulsan, South Korea; 2 Department of Pediatrics, Chung-Ang University Hospital, College of Medicine, Chung-Ang University, Seoul, South Korea; 3 Department of Pediatrics, Konkuk University Medical Center, Konkuk University School of Medicine, Seoul, South Korea; 4 Department of Pediatrics, Haeundae Paik Hospital, Inje University College of Medicine, Pusan, South Korea

**Keywords:** jaundice, neonatal intensive care unit, phototherapy, transcutaneous bilirubin

## Abstract

**Background:**

To confirm the accuracy of transcutaneous bilirubin (TcB) in the neonatal intensive care unit both with and without phototherapy, and compare forehead and sternum as the TcB assessment site.

**Methods:**

We simultaneously assessed the total serum bilirubin (TSB) and TcB at the forehead and sternum, using a JM-103 bilirubinometer. We analyzed the correlation between the TSB and TcB assessed at the forehead and sternum, with measurements classified as ‘without phototherapy’ (before phototherapy and > 24 hours after phototherapy discontinuation) and ‘with phototherapy’ (after 24 hours of phototherapy).

**Results:**

There were 1,084 paired forehead and sternum TcB measurements, with the corresponding TSB measurement, from 384 infants. Their mean gestational age of 35.4 ± 3.2 weeks (62% were preterm) and a mean birth weight of 2434 ± 768 grams, and TSB was 6.61 ± 3.56 mg/dL. Without phototherapy, TcB values at the forehead and sternum were correlated well to the TSB value (*r* = 0.925 and 0.915, respectively). With phototherapy, TcB values at the forehead and sternum were significantly correlated with the TSB value, but TcB at the forehead (*r* = 0.751) was a better match to the TSB than was TcB at the sternum (*r* = 0.668). Additionally, Bland-Altman plots showed a greater degree of underestimation of the TSB by TcB at the sternum with phototherapy.

**Conclusions:**

TcB was more accurate in infants not receiving phototherapy. During phototherapy, it is better to assess TcB at the forehead rather than at the sternum.

## Background

The American Academy of Pediatrics (AAP) recommends screening for jaundice and its risk factors before newborns leave the hospital. In healthy newborns, transcutaneous bilirubin (TcB) assessment has been widely used for screening, as it does not require blood sampling and provides a quick result. By contrast, total serum bilirubin (TSB) assessment requires blood sampling, which is an invasive and painful procedure. However, the accuracy of TcB in infants receiving phototherapy is lower due to skin bleaching [1–4]. Furthermore, there is doubt regarding its accuracy under phototherapy because phototherapy converts bilirubin through the skin. Although several recent studies regarding TcB assessment after the application of phototherapy have reported its good reliability, other studies have reported a low reliability. This difference in results could be due to differences in assessment sites, target patients, presence of a skin patch, and devices used. Various assessment sites have been used, including the forehead, sternum, interscapular space, hipbone, and lower abdomen, with the forehead and sternum as the most frequently used sites.

In the present study, we aimed to confirm the accuracy of TcB under phototherapy, as well as determine whether the forehead or sternum as the assessment site results in a more accurate TcB assessment under phototherapy in sick infants admitted to the neonatal intensive care unit (NICU).

## Methods

The study was performed via retrospective chart review in the NICU of the Ulsan University Hospital, a tertiary referral center from September 2018 to August 2019. TcB values assessed simultaneously at the forehead and sternum by a physician (Lim G.) within one hour before or after blood sampling for TSB analysis using a Minolta Air Shields Jaundice Meter (JM-103, Dräger Medical, Lübeck, Germany). TcB measurements obtained before the initiation of phototherapy or more than 24 hours after phototherapy discontinuation were classified as ‘without phototherapy’. TcB measurements obtained under phototherapy, which usually lasted more than 24 hours, were classified as ‘with phototherapy’. Demographic data, including the gestational age, birth weight, sex, race, postnatal age and mode of delivery, were collected. Blood for TSB measurements was drawn by vessel puncture; TSB levels were measured in the hospital’s clinical chemistry laboratories using the Roche cobas c702 analyzer (Roche Diagnostics International Ltd., Rotkreuz, Switzerland). Patients were treated with continuous phototherapy using a standard LED phototherapy unit, either the Medix® MediLED™ (Natus Medical Incorporated, Pleasanton, CA, USA) or the Giraffe® Blue Spot PT Lite™ (GE Healthcare, Chicago, IL, United States). This study was approved by the institutional review board of Ulsan University Hospital (#2020-03-032). The datasets used and/or analysed during the current study available from the corresponding author on reasonable request.

The association between TcB and TSB was evaluated by linear regression analysis and Bland-Altman plots, and compared among four measurement groups: forehead TcB without phototherapy, sternum TcB without phototherapy, forehead TcB with phototherapy, and sternum TcB with phototherapy. *P* value less than 0.05 was considered significant. No adjustment was made for multiple comparisons. All statistical analyses were performed using SPSS (ver. 21, IBM Co., Armonk, NY, USA).

## Results

A total of 415 infants were admitted to the NICU; 384 infants with 1,084 paired forehead and sternum TcB and TSB values were included. The other infants (n = 31) were excluded, as they had no TcB values corresponding with TSB. There were 861 measurements classified as ‘without phototherapy’ and 223 measurements classified as ‘with phototherapy’. The characteristics of included infants are shown in Table 1. The TSB was 6.61 ± 3.56 mg/dL (6.11 ± 3.42 without, and 8.54 ± 3.43 with phototherapy), while the forehead TcB was 6.19 ± 3.97 mg/dL (6.12 ± 4.00 without, and 6.50 ± 3.86 with phototherapy), and the sternum TcB 5.85 ± 3.92 mg/dL (6.24 ± 3.97 without, and 4.34 ± 3.30 with phototherapy).

**Table 1:** Characteristics of infants

Characteristics	
Gestational age (weeks)	35.4 ± 3.2
<32	49 (12.8)
32–36	189 (49.2)
37–	146 (38.0)
Birth weight (g)	2434 ± 768d
< 1500	51 (13.3)
1500–2499	157 (40.9)
2500–	176 (45.8)
Male	210 (54.7)
Cesarean section	253 (65.9)
Postnatal age (days)	10.7 ± 12.7
No of paired TSB TcB values	2 (1–4)
< 1500	6 (4–7)
1500–2499	3 (2–4)
2500–	2 (1–2)

Data shown as mean ± SD, n (%), and median (interquartile range)

**Fig. 1:**
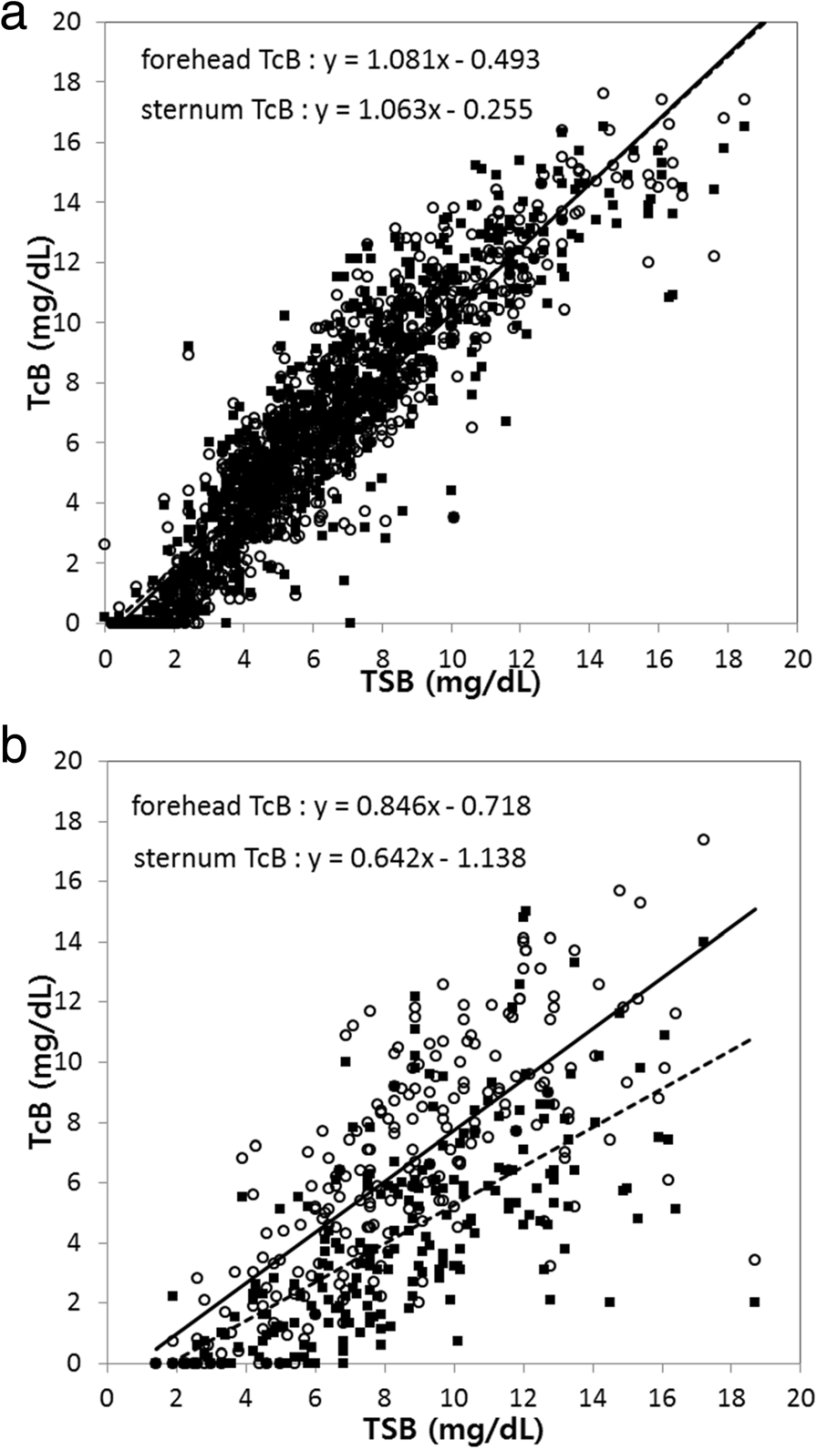
Correlation between TSB and TcB. **a** Correlation between TSB and TcB values without phototherapy. **b** Correlation between TSB and TcB values with phototherapy (○ and solid line: TcB at the forehead, ■ and dashed line: TcB at the sternum). The x-axis shows the TSB value and the y-axis shows the TcB value. TcB, transcutaneous bilirubin; TSB, total serum bilirubin

On linear regression analysis without phototherapy, the forehead (y = 1.08x–0.49, *r* = 0.925, *P* < 0.001) and sternum (y = 1.06x–0.26, *r* = 0.915, *P* < 0.001) TcB values were both well matched to the corresponding TSB value (Fig. 1-a). With phototherapy, the forehead TcB (y = 0.85x − 0.72, *r* = 0.751, *P* < 0.001) and sternum TcB values (y = 0.64x–1.14, *r* = 0.668, *P* < 0.001) remained well matched to the corresponding TSB value; however, the correlation coefficients were lower than those obtained without phototherapy. Furthermore, with phototherapy, the forehead TcB was better matched to the corresponding TSB value than the sternum TcB (Fig. 1-b).

**Fig. 2:**
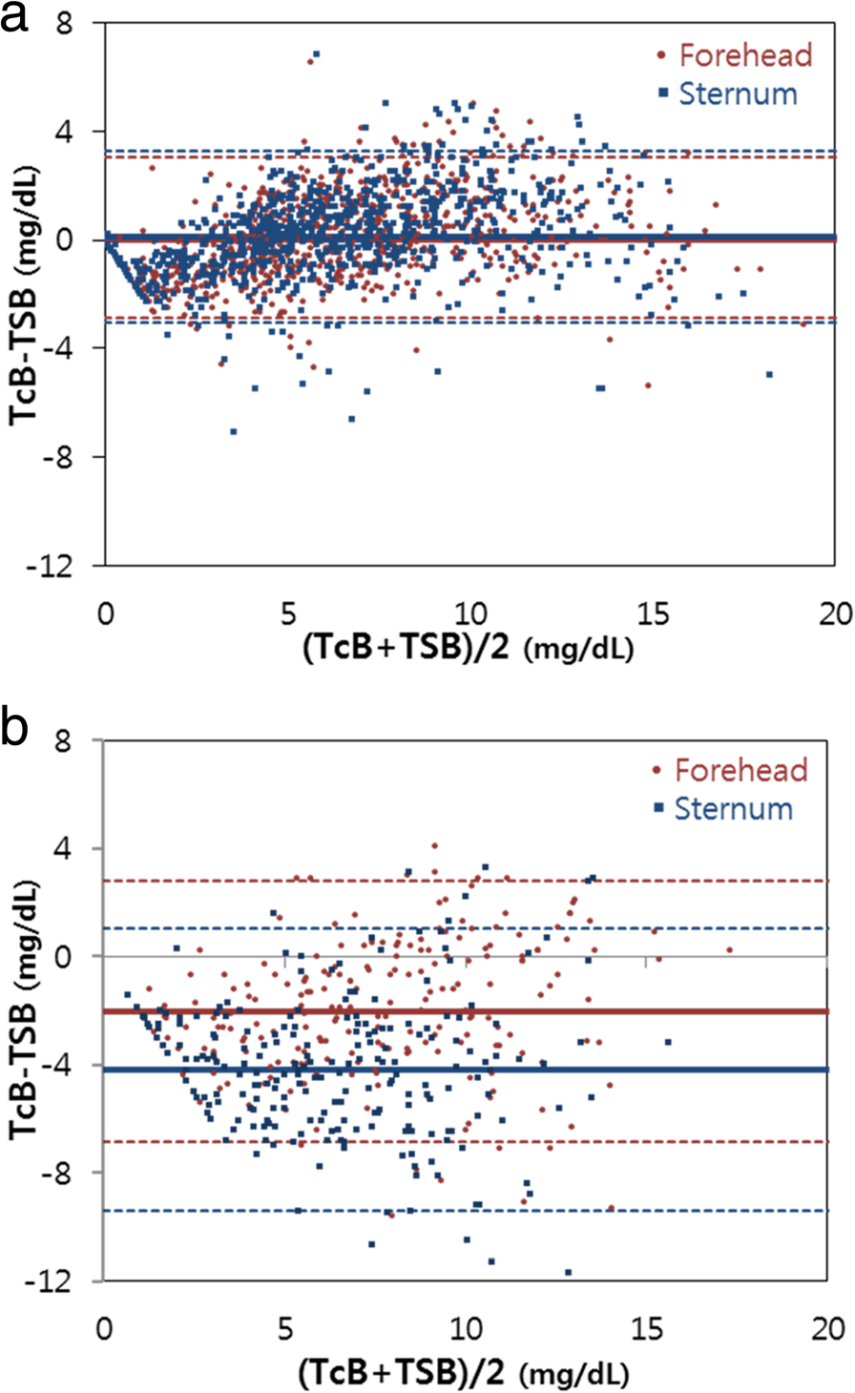
Bland-Altman plots of TSB and TcB. Bland-Altman plots of without phototherapy (**a**), and with phototherapy (**b**). The x-axis shows the mean TSB and TcB (mg/dL) values, and the y-axis shows the difference between TSB and TcB (mg/dL) values. The red colour represents forehead values and blue colour represents sternum values. The solid horizontal line represents the mean difference; the dashed lines represent the 95% confidence interval. TcB, transcutaneous bilirubin; TSB, total serum bilirubin

Bland-Altman plots of the difference between the TcB and TSB values versus the average of the TcB and TSB values are shown in Fig. 2. Without phototherapy, the mean difference between forehead TcB and TSB values was 0.00 ± 1.54 mg/dL, while the mean difference between sternum TcB and TSB values was 0.13 ± 1.62 mg/dL. With phototherapy, the mean difference between forehead TcB and TSB values was − 2.00 ± 2.46 mg/dL, and the mean difference between sternum TcB and TSB values was − 4.17 ± 2.66 mg/dL. Thus, the Bland-Altman plots showed a greater degree of underestimation (negative mean bias) of the TSB level by the TcB at the sternum than by the TcB at the forehead, with phototherapy.

## Discussion

Our study confirmed that TcB assessment in NICUs is accurate, more so in infants who are not receiving phototherapy than in infants who are; additionally, under phototherapy, the forehead was the better site for TcB assessment.

In full-term infants not receiving phototherapy, TcB is considered to be an established test and a reasonable alternative to TSB [5, 6]. However, there is some doubt regarding the use of TcB as a complete replacement for TSB [7], especially in preterm and low birth weight infants [8]. Although some studies have reported that TcB is less accurate in preterm infants than in full-term infants, a greater number of studies have reported that TcB is an accurate and reasonable alternative to TSB in preterm infants [9–11]. Furthermore, many recent reports have verified the accuracy and usefulness of TcB in preterm and low birth weight infants [12]. Thus, the present study utilized inclusion criteria that allowed a wide range of infants, in terms of gestational age and birth weight, to participate; all infants who were admitted to our NICU, and thus had risk factors for jaundice by the Bhutani normogram [13] were included. Even with the inclusion of admitted NICU infants, the present study found that TcB had good accuracy, comparable to that in other studies. Specifically, in infants not receiving phototherapy, the present study showed a good correspondence between TcB and TSB values by linear regression and Bland-Altman plots. Thus, TcB is valuable in estimating the TSB and checking for jaundice in NICU.

The use of TcB assessment has expanded to include infants receiving phototherapy. There are many recent reports regarding the accuracy and usage of TcB under phototherapy [12, 14]. Significant correlations between TSB and TcB values under phototherapy have been reported, with coefficients ranging from 0.7 to 0.9. We obtained correlation coefficients of 0.751 and 0.668 for the forehead and sternum, respectively, which are similar to those in previous reports, but are much lower than those obtained in infants who are not receiving phototherapy. The basic mechanism of the transcutaneous bilirubinometer (JM-103) is the measurement of the yellowness of the subcutaneous tissue by short and long optical paths. Therefore, reduced TcB accuracy under phototherapy is natural, and this should be considered in clinical applications. For example, TcB assessment under phototherapy could suggest the trend in TSB, reducing the number of blood tests, and phototherapy cessation could be determined by considering the error range. In the present study, TcB assessment under phototherapy tended to underestimate the TSB value, but some overestimation also occurred. TcB under phototherapy was usually assessed more than 24 hours after the initiation of phototherapy, and we did not consider the duration of phototherapy, which may influence the accuracy of TcB. Previous studies have reported that the degree of underestimation of the TSB by TcB is largest during the first 8 hours of phototherapy, with the difference gradually returning to pre-treatment values [15]. The duration of phototherapy also influences accuracy, with a longer duration resulting in less underestimation of the TSB level by TcB. We analyzed TcB measurements more than 24 hours after the initiation of phototherapy as a group, obtaining a good correlation coefficient and less underestimation than that in previous reports.

We found that, in infants not receiving phototherapy, TcB correspondence with the TSB level did not differ according to assessment site (forehead vs. sternum). However, in infants receiving phototherapy, TcB correspondence with the TSB level differed according to assessment site. Specifically, TcB at the forehead was better matched to the corresponding TSB measurement, and had less underestimation, compared to that for TcB at the sternum. Usually, TcB is assessed at the forehead or sternum. Without phototherapy, the JM-103 manual recommends assessing TcB at the sternum, and the sternum is known as the best site for the JM-103 device [12, 16, 17]. However, the recommendation might need to be different with phototherapy; most studies have reported that, under phototherapy, forehead TcB is better than sternum TcB. In the present study, the correlation coefficients with phototherapy were 0.751 at the forehead and 0.668 at the sternum. These results may be due to not only craniocaudal progression characteristics of jaundice itself [18], but also a better correlation with the TSB level for forehead TcB than for sternum TcB, as shown in Bland-Altman plots. Studies comparing the forehead and sternum as assessment sites under phototherapy are rare. In real clinical practice, we have mostly used the forehead and sternum for TcB assessment. Some reports have used other sites, such as the interscapular space, hipbone, abdomen [10], and patch-covered skin; however, these sites are not commonly used in real clinical practice. Additionally, although TcB assessment at covered skin (*r* = 0.80) has been shown to be much better than TcB assessment at uncovered skin, the use of a patch is not convenient [2, 19]. Therefore, mirroring the clinical situation, it is important and helpful to compare forehead and sternum TcB under phototherapy. This study was planned based on this concept and concluded that it is better to assess TcB at the forehead, rather than the sternum, in infants receiving phototherapy.

The present study has some limitations. First, TcB with phototherapy was not subdivided according to the duration of phototherapy. Second, the study participants were sick infants who required NICU admission. We did not adjust for confounding factors of gestation, postnatal age, and sickness (e.g. shock leading to less perfusion). Thus, the present results should be applied to NICU infants. Third, the TSB level was not very high, as the infants were under close observation and the treatment threshold is different for preterm and ill infants. And last, it would be better if it could be used for decision making test to start or stop phototherapy.

## Conclusions

TcB had a fair reliability in NICUs, and was more accurate in infants not receiving phototherapy than in infants receiving phototherapy. In addition, during phototherapy, it is better to assess TcB at the forehead, rather than at the sternum.

## Data Availability

All data generated or analyzed during this study are included in this published article.

## Abbreviations

AAP: American Academy of Pediatrics
NICU: Neonatal intensive care unit
TcB: Transcutaneous bilirubin
TSB: Total serum bilirubin
